# Patients’ Perspectives on Artificial Intelligence in Dentistry: A Controlled Study

**DOI:** 10.3390/jcm11082143

**Published:** 2022-04-12

**Authors:** Esra Kosan, Joachim Krois, Katja Wingenfeld, Christian Eric Deuter, Robert Gaudin, Falk Schwendicke

**Affiliations:** 1 Charité—Universitätsmedizin Berlin, Corporate Member of Freie Universität Berlin and Humboldt-Universität zu Berlin, Department of Oral Diagnostics and Digital Health and Health Services Research, Aßmannshauser Str. 4-6, 14197 Berlin, Germany; joachim.krois@charite.de (J.K.); falk.schwendicke@charite.de (F.S.); 2 Charité—Universitätsmedizin Berlin, Corporate Member of Freie Universität Berlin and Humboldt-Universität zu Berlin, Department of Psychiatry and Psychotherapy, Hindenburgdamm 30, 12200 Berlin, Germany; katja.wingenfeld@charite.de (K.W.); christian.deuter@charite.de (C.E.D.); 3 Charité—Universitätsmedizin Berlin, Corporate Member of Freie Universität Berlin and Humboldt-Universität zu Berlin, Department of Oral and Maxillofacial Surgery, Hindenburgdamm 30, 12200 Berlin, Germany; robert-andre.gaudin@charite.de

**Keywords:** artificial intelligence, communication, dental diagnosis, machine learning, patients, trust

## Abstract

Background: As artificial intelligence (AI) becomes increasingly important in modern dentistry, we aimed to assess patients’ perspectives on AI in dentistry specifically for radiographic caries detection and the impact of AI-based diagnosis on patients’ trust. Methods: Validated questionnaires with Likert-scale batteries (1: “strongly disagree” to 5: “strongly agree”) were used to query participants’ experiences with dental radiographs and their knowledge/attitudes towards AI as well as to assess how AI-based communication of a diagnosis impacted their trust, belief, and understanding. Analyses of variance and ordinal logistic regression (OLR) were used (*p* < 0.05). Results: Patients were convinced that “AI is useful” (mean Likert ± standard deviation 4.2 ± 0.8) and did not fear AI in general (2.2 ± 1.0) nor in dentistry (1.6 ± 0.8). Age, education, and employment status were significantly associated with patients’ attitudes towards AI for dental diagnostics. When shown a radiograph with a caries lesion highlighted by an arrow, patients recognized the lesion significantly less often than when using AI-generated coloured overlays highlighting the lesion (*p* < 0.0005). AI-based communication did not significantly affect patients’ trust in dentists’ diagnosis (*p* = 0.44; OLR). Conclusions: Patients showed a positive attitude towards AI in dentistry. AI-supported diagnostics may assist communicating radiographic findings by increasing patients’ ability to recognize caries lesions on dental radiographs.

## 1. Introduction

Artificial intelligence (AI), in particular machine learning (ML), has become increasingly popular to support medical diagnostics [1]. Recent AI applications, for example, in dermatology [2], facilitate the detection of diseases with an accuracy comparable to that of experts [3,4]. Furthermore, in dentistry, AI is increasingly adopted, mainly for the analysis of dental imagery such as radiographs [5] or intraoral scans but also to support decision making and predicting disease pathways [6].

Dental caries is the most frequent prevalent human condition worldwide, with more than two billion cases [7]. Preventing caries and arresting early caries lesions is a main goal of contemporary dentistry. For detecting proximal caries lesions, dentists rely not only on visual and tactile examination of the teeth [8] but also on bitewing radiography [9]. The detection and evaluation of caries lesions on bitewing radiographs, however, has limited reliability and validity. Schwendicke et. al, for example, observed low sensitivities in the range of 0.24–0.42 [10]; significant diagnostic differences between dentists and a significant proportion of false positive or negative findings have been reported [10]. Consequently, recent studies used AI, specifically ML, to detect caries lesions on bitewings and found that the accuracy (mainly the sensitivity) of AI/ML was significantly higher than that of an independent dentist [11,12]. Latest research indicated that intraoral scans using near-infrared light reflection (NILR) may also be more sensitive for caries detection than classical bitewing radiography [13].

The adoption of AI into dental care may depend on a wide range of factors, one of them being dental patients: AI applications may increase their trust in dental diagnosis and foster their understanding of their dentists’ decision [14] or may stir worries and reservations. In other fields, such as radiology, qualitative [15] and quantitative [16] approaches were used to map patients’ understanding and attitudes towards AI. For example, when asking patients who had received computer tomography (CT) scans of their chest or abdomen how they would react towards this CT being analyzed by an AI only, six relevant themes were identified [15]: (1) “Distrust and accountability”: Patients trusted humans more than the AI even if both were equally accurate. Moreover, patients needed to be convinced of the AI’s superiority by research data. (2) “Procedural knowledge”: Patients wanted to understand how the AI would be implemented in order to assess their CT and what role the AI and the medical staff would have. (3) “Competence”: Patients were unsure of an AI’s ability to read and interpret CT scans compared to the experience of a radiologist. They would prefer if the AI was applied as a second opinion instead. (4) “Efficiency”: Patients believed that AI works faster and more efficiently than a human, therefore reducing waiting time and costs. (5) “Personal interaction”: Receiving the results of their CTs from a doctor was important to patients. They needed to interact with another human when receiving the findings of their scans. (6) “Accountability”: For human mistakes, doctors can be held accountable, while patients wondered who will be held accountable for an AI’s mistakes [15].

However, AI in dentistry is not supposed to be used on its own but as a “second opinion” and support tool [6]. Moreover, dental patients may be more familiar with possible diagnoses on dental radiographs (such as caries lesions) than patients receiving chest or abdomen CTs (given most patients attending the dentist regularly), and dental patients may also be used to have their radiographic findings explained by their dentist. AI can hence be expected to influence patients’ trust and understanding of dental radiographic diagnoses differently than in other fields.

In the present study, we aimed to evaluate patients’ knowledge of and attitude towards AI in dentistry in an observational study as well as the impact of AI-based diagnosis and communication on their trust in a nested, controlled investigation. We hypothesized that AI-based communication increased patients’ understanding, belief, and trust in their dentists’ radiographic diagnosis.

## 2. Materials and Methods

### 2.1. Study Design

This observational study was approved by the ethics committee of the Charité–Universitätsmedizin Berlin (application number: EA4/251/20). Pilot-tested questionnaires were used for a cross-sectional assessment of participants’ experiences with dental radiographic diagnosis, their prior knowledge of, and their attitudes towards AI in general and especially in dentistry. For those patients who did not understand the meaning of AI, a short introduction into the field was given before further applying the questionnaire: AI is “the theory and development of computer systems able to perform tasks normally requiring human intelligence, such as visual perception, speech recognition, decision-making, and translation between languages” [17].

**Figure 1:**
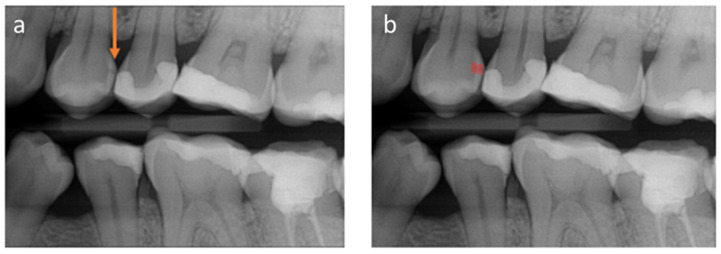
The radiographs shown in the vignette investigation. (**a**) Radiograph with a caries lesion; the orange arrow indicates the presence of the caries lesion (standard communication/control). (**b**) The same radiograph, with the caries lesion being indicated by an AI-generated pixel blob in red (AI-based communication).

To explore the impact of AI-based communication on patients’ understanding of and trust into the diagnosis, a controlled investigation was conducted in addition. We used two vignettes to simulate two scenarios: one (control) where patients were shown conventional radiographs, and the caries lesion was highlighted by an arrow (similar to what would be done clinically without AI), and the next was one where an AI-based communication of the detected caries lesion was employed (test) (Figure 1). The applied program, dentalXrai, is a fully convolutional neural network that is able to label findings in radiographs in a pixel-wise manner. In the first vignette (control), the following introduction was made: “Imagine that your dentist shows you the above radiograph and explains to you that you can see several fillings (white) and an advanced caries lesion (orange arrow). Your dentist will explain to you that this caries lesion needs to be removed and replaced with a filling. This will come with costs of around EUR 80.” Afterwards, the patients were asked to assess their ability to see and understand the diagnosis (Table 1). In the second vignette (test), we showed the conventional radiograph and, additionally, the same radiograph with an AI-generated coloured overlay (Figure 1). This time, the following introduction was given: “Imagine that your dentist shows you the radiograph on the left and explains that you can see several fillings and an advanced caries lesion (orange arrow). In addition, your radiograph is evaluated by a computer program that works with AI. The program is able to identify caries, inflammation of the roots, bone loss, etc., self-sufficiently and independently of the dentist with one click. These pathologies are then marked in colour by the program. In the right radiograph, the program marks the spot where the caries lesion can be seen in red. Your dentist will explain to you that he/she agrees with the diagnosis of the program and recommends a removal of the caries lesion and replacement with a filling. This will come with costs of around EUR 80”. Again, participants were asked to evaluate the same statements (Table 1). Evaluation was performed using a Likert scale (1 = strongly disagree, 2 = disagree, 3 = neither agree nor disagree, 4 = agree, 5 = strongly agree).

To assess potential confounding variables, we evaluated the personality of the participants using the Big-Five-Inventory-10 (BFI-10) [18]. The BFI-10 was developed to help comprehend the five main dimensions of a personality, namely “extraversion”, “tolerance”, “conscientiousness”, “neuroticism”, and “openness” [18]. For this purpose, each participant rated ten statements about their personality using a five-level rating scale (1 = strongly disagree, 2 = disagree a little, 3 = neither agree nor disagree, 4 = agree a little, 5= strongly agree). Moreover, the Patient Health Questionnaire for Depression and Anxiety (PHQ-4) [19] was used to determine whether subjects suffered from anxiety disorders or depression at the time they participated in the survey. An increased PHQ-4 was associated with “functional impairment, disability days, and healthcare use” [19]. Scores are determined by adding together the scores of each of the four items and rated as normal (0–2), mild (3–5), moderate (6–8), and severe (9–12). A total score ≥ 3 for the first two questions suggests anxiety and for the last two questions depression [19].

Moreover, patients were asked to state their exact birthdates, gender, and level of education written in their own words.

### 2.2. Setting and Participants

**Figure 2:**
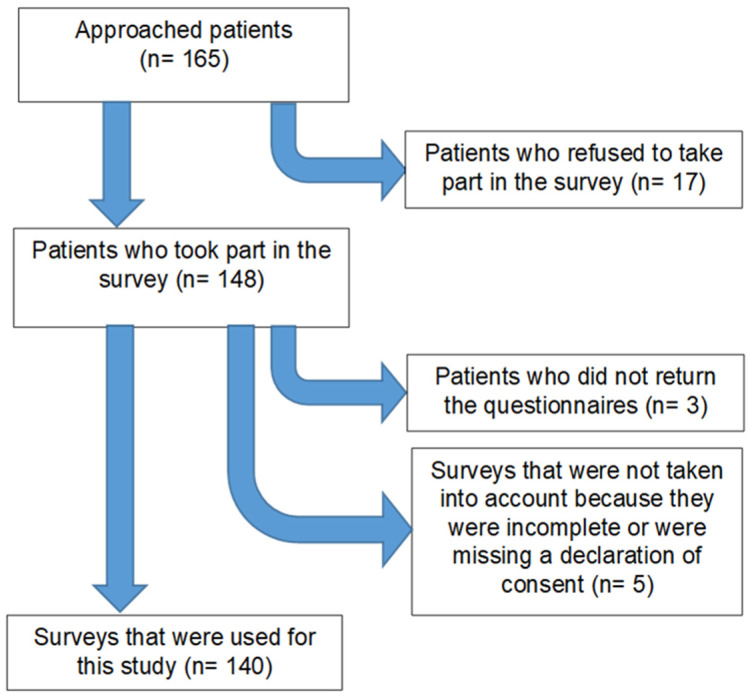
Flowchart describing the process of patient acquisition for this survey.

In total, 140 patients from the Charité–Universitätsmedizin Berlin dental clinic were randomly recruited for this study between February and March 2021. The participants flow is shown in Figure 2. All patients aged > 18 years with sufficient German proficiency who visited the emergency service or were patients in one of the clinical student courses were asked to take part in this survey. Patients concluded the survey in an average of 15–20 min during a waiting period or after they received treatment. Each participant was introduced to the general topic of the questionnaire by a dentist and was given the opportunity to ask questions before and during the completion of the survey. Patients returned the surveys before leaving the clinic.

### 2.3. Variables

We chose to evaluate the outcomes based on the following variables: “see” (I can see the caries on the radiograph), “believe” (I believe in my dentist’s decision), “trust” (I trust my dentist’s decision), “understand” (I understand the need for treatment), “feel informed” (I feel adequately informed), and “feel unable” (I feel unable to make a decision on my own”). As confounders, we chose age, sex, education, and employment. The PHQ-4 and the BFI-10 scores were chosen as co-variates.

### 2.4. Bias

While recruiting patients from a university clinic introduced the risk of selection bias, we sampled inclusively from the attending patient pool, with the only exclusion criteria being age and language proficiency (which both may introduce bias), as explained. Further bias by patients responding according to expected desirability, etc., could not be prevented.

### 2.5. Sample Size

The number of cases to be sampled was determined based on the primary outcome, “see” (I can see the caries on the radiograph). The sample size was estimated assuming the use of the McNemar test, with approximately 30% of the individuals being able to recognize caries on a conventional radiograph without AI support. The AI application was assumed to increase this to 50%. With a power 1-beta = 0.80 and alpha = 0.05 (two-sided) and an assumed 30% discordant pairs, this resulted in a sample size of 139 subjects.

### 2.6. Quantitative Variables

For statistical interpretation, the mean values of the Likert scale assessment of all questions were compared between different groups of age, sex, education, and employment. Additionally, an ordinal logistic regression (OLR) was performed to assess the impact of AI-based communication using the difference in Likert scale answers (1–5) with versus without AI as outcome metric.

### 2.7. Statistical Methods

Data analysis was conducted using SPSS for Windows 26.0 (IBM, Armonk, NY, USA). The *p*-value was set at 0.05. Mean value comparisons and OLR analyses were conducted. Surveys with missing data were excluded. No imputation was performed.

## 3. Results

Out of the 165 patients approached, 17 patients were not able to participate in our survey (Figure 2), mainly due to a lack of time. Of the remaining 148, 8 surveys were excluded, as they were incomplete, or the informed consent was missing. The final sample (*n* = 140) was aged between 18 and 84 years (mean ± standard deviation: 52 ± 17 years). A total of 69 (49.3%) of the respondents were male, while 71 (50.7%) were female. Overall, 64 (45.7%) had a university degree, while 76 (54.3%) did not. One-hundred and three (73.5%) of the interviewed patients were currently employed, while twelve (8.6%) were currently unemployed, eleven (7.9%) were students, and fourteen (10%) were retired. The overall PHQ-4 was 2.6 ± 2.3 (min./max.: 0.0/11.0), while the sub-scores for depression and anxiety were 1.3 ± 1.3 (min./max. 0.0–6.0) and 1.3 ± 1.4 (min./max.: 0.0–7.0), respectively. In detail, 12.9% of patients revealed a mild and one person a moderate form of depression, while 10% revealed mild and 2.9% moderate anxiety. Male participants revealed significantly higher PHQ-4 scores (*p* < 0.05; ANOVA).

A total of 97.9% of the participants remember having had a radiograph of their teeth or jaw taken in the past, and in 65.0%, caries had been detected on these radiographs at least once. Next, 88.6% of the participants had experienced caries; 95.0% of the acquired patients had heard about “artificial intelligence”, and 90.7% had a general understanding of it.

Overall, patients were convinced that “AI is useful” (4.19 ± 0.8) and did not fear the usage of AI in general (2.23 ± 1.0) and especially not in dentistry (1.65 ± 0.8). It became apparent that patients valued being informed of their health status (4.75 ± 0.6) and that they wanted to understand the results of their radiographs (4.56 ± 0.7) (Table 2). When comparing the mean Likert values, using AI-based communication significantly increased patients’ ability to see the caries lesion (*p* < 0.0005), while there were no significant differences in the other assessed dimensions (e.g., trust, etc.) (Table 1).

OLR was conducted to assess how covariates moderated the effect of the AI-based communication on the different outcomes (Table 3). Younger patients (aged < 52 years) and anxious ones benefitted significantly more from the AI-based communication when it came to seeing the lesion (mean Likert scale difference (95% CI) 1.12 (0.38/1.86) and 0.99 (0.13/1.86), respectively). Moreover, individuals with university education saw lesions significantly better when AI-based communication was used (0.90 (0.21/1.59)). Students showed an increased belief into their dentists’ diagnosis when AI-based communication had been employed (1.98 (0.34/3.62)), while extroverted patients showed the opposite (−0.38 (−0.6/−0.08)). Neuroticism was found associated with an increased benefit of AI-based communication on trust in the diagnosis (0.48 (0.01/0.96)), and openness led to patients feeling more informed of their dental health status (0.64 (0.05/1.24)).

## 4. Discussion

The present study assessed the experiences and attitudes of patients with/towards AI for a specific use-case, dental radiographic analysis and the impact of AI-based communication of findings on these radiographs. Our null-hypothesis was that AI-based communication increased patients’ understanding, belief, and trust in their dentists’ radiographic diagnosis. We partially accept this hypothesis; overall, neither belief, understanding, nor trust were significantly affected; however, students showed a significantly improved belief in their dentists’ diagnosis, and patients with increased neuroticism appeared to be more trusting.

We also observed an increase in detectability (“see”). One can speculate that an increased ability to see the caries lesion on the shown radiograph subsequently increased the belief in the dentist’s diagnosis and the suggested therapy. It is possible that the interviewed patients were unable to understand the causality between the shown caries lesion and the need for treatment because patients naturally lack the required domain-specific knowledge. It should be noted that standard bitewing radiography is not the only diagnostic tool to detect caries, and detectability varies between different approaches, e.g., 2D vs. 3D radiography [20].

The trust in a dentist’s decision was high in both conventional communication and AI-based communication; patients generally trusted their dentist. Similar results regarding trust in dentists were found in a study surveying Australian patients [21]. According to Mayer et al., interpersonal trust is a human belief based on benevolence, integrity, and ability [22]. The main difference between interpersonal trust (e.g., between dentist and patient) and trust in technical systems (e.g., into an AI-based software) is a lack of intentionality, which is an important factor to honesty and benevolence [23]. Moreover, trust in machines relies on their reliability and functionality [24]. As described by Thom et al., “Trust in another person refers to an expectation that the other person will behave in a way that is beneficial, or at least not harmful, and allows for risks to be taken based on this expectation.” [25]. This can be transferred to our scenario; trust in AI is the expectation that this technology will provide beneficial recommendations for a patient’s health, allowing for risks to be taken based on this expectation. Previously, it was proven that human decisions in medicine were preferred over that of AI applications despite their proven superiority [16,26,27]. This does not necessarily align with the findings of our study. A possible explanation could be that humans prefer to interact with other humans compared to interacting directly with an AI application [28]. Our study held a scenario that allowed for human contact; moreover, the responsibility lay solely with the dentist, possibly bypassing this phenomenon.

A range of socio-demographic factors were found to modify the impact of AI as indicated by the ORL; younger, more educated patients or students benefitted more from the AI-based communication. A previous study regarding skeletal radiography [29] revealed concordant results. Elderly patients are generally more likely to be sceptical towards technology in health care [30] and are often fearful of being dependent on technology in the context of health and caregiving [31]. Notably, in our sample, 45.7% of the participants had a university degree, while the same level of education in the whole German population of 2020 is only 18.5% [32]. Individuals with an academic background were hence overrepresented in our survey, which should be borne in mind when interpreting our results.

The average PHQ-4 revealed that the personality profile was in the expected range of—by large—psychologically healthy individuals [33]. It was shown that anxious individuals have slower perceptual processing [34], which could explain why anxiety moderated the effect of the AI-tool, and anxious patients benefitted more from it than non-anxious ones when it came to seeing the caries on a radiograph. Neurotic people feel physically and psychologically impaired; their pessimism and anxiety affects their health negatively [35]. Moreover, neuroticism is negatively associated with interpersonal trust [36]. Therefore, the additional, objective diagnostic AI tool such as that used in this study may have particularly increased trust in these patients. Extroverted individuals seek the company of humans and are open to experiences and social contacts [36] and may hence benefit less from the AI tool. In contrast, openness positively influenced the effect of the AI-tool when it came to patients feeling informed of their health status, which may be linked to such patients generally appreciating additional sources of information (such as an AI tool).

The present study has a number of strengths and limitations. First, it is one of few studies focusing on the attitudes of patients towards medical and specifically dental AI and also used a controlled design to assess if AI was beneficial for patient–provider communication. The nested controlled study, however, employed hypothetical scenarios only, and participants were asked to imagine having the described disease (caries). There is always the question of how well our findings would replicate in real-life situations. However, only a small number of participants never had a caries lesion before, so it can be assumed that most patients were able to empathize with the given scenario. Second, the study was conducted during a time of high surge of COVID-19 cases in all of Germany. Vulnerable patients were asked to postpone their appointments and only seek help in our facility if absolutely necessary. Given these circumstances, it is likely that the participants’ spectrum was skewed towards patients with less comorbidities and better health; i.e., representativeness of our sample may not be fully given. Moreover, the sample size was limited, and while it was sufficient to detect significant differences between scenarios, larger samples may allow for more detailed subgroup analyses. Last, there are only few validated instruments available to assess patients’ attitudes towards technology in healthcare and specifically AI, which is why we relied on existing (but not necessarily validated) questionnaires from peer-reviewed studies [15,16] or non-peer-reviewed surveys [37]. Future studies should attempt to validate these.

## 5. Conclusions

Although patients’ trust was not affected by the application of an AI-based diagnosis, patients showed a positive attitude towards AI in dentistry. AI-supported diagnostics may assist communicating radiographic findings, since the investigated AI-based instrument was able to increase patients’ ability to recognize caries lesions on dental radiographs. Therefore, dentists should use AI-based tools, if available, to improve patient–provider communication. In the future, AI-based instruments will likely gain importance in clinical dentistry. Our study revealed great potential in patients’ acceptance of such tools in daily practice.

## Figures and Tables

**Table 1:** Findings of the vignette-based controlled investigation. The mean (standard deviation) Likert-scale values (from 1—strongly disagree to 5—strongly agree) are shown. Bold values indicate statistically significant differences (*p* < 0.05/Wilcoxon).

Statement	Variable	Group	Mean (SD)
“I can see the disease (caries) on the radiograph”.	See	Control: (no AI)	**2.8 (1.3)**
		AI-based communication	**3.5 (1.3)**
“I believe that my dentist can make a correct diagnosis (caries) based on radiograph and suggest an appropriate therapy (filling)”.	Believe	Control: (no AI)	4.3 (0.8)
		AI-based communication	4.4 (0.7)
“I understand that the disease (caries) seen on the radiograph needs treatment”.	Understand	Control: (no AI)	4.3 (0.9)
		AI-based communication	4.4 (0.8)
“I trust the decision my dentist has made based on the radiograph”.	Trust	Control: (no AI)	4.4 (0.8)
		AI-based communication	4.5 (0.6)
“I feel adequately informed about the condition (caries) and the need for a therapy (filling)”.	Feel informed	Control: (no AI)	3.9 (1.1)
		AI-based communication	4.2 (0.9)
“I have the feeling that I cannot make a decision on my own, and I feel at the mercy of my dentist”.	Feel unable	Control: (no AI)	2.7 (1.3)
		AI-based communication	2.7 (1.3)

**Table 2:** Descriptive analysis of patients’ attitudes towards AI and, specifically, its application in dentistry. The mean (standard deviation) Likert-scale values (from 1—strongly disagree to 5—strongly agree) are shown.

Statements	Mean (SD)
“AI is useful”	4.2 (0.8)
“In principle, I would prefer to rely on humans rather than robots or AI.”	3.4 (1.0)
“I have security concerns about using AI.”	2.7 (1.0)
“AI makes life easier.”	3.8 (0.8)
“I believe AI improves people’s quality of life.”	3.6 (0.9)
“I am scared of the topic and its consequences”	2.2 (1.0)
“I would base the choice of my dentist on whether he/she uses AI-based assistance systems or not.”	2.5 (1.2)
“If my dentist doesn’t use AI-based assistance systems, I would switch to another dentist.”	2.0 (1.0)
“The use of AI in dentistry scares me.”	1.7 (0.8)
“I think that AI will support dentists’ diagnostics in the future.”	4.2 (0.8)
“AI can prevent incorrect treatment.”	3.7 (0.8)
“I can have more confidence in a dentist’s diagnosis when it’s assisted by AI.”	3.4 (1.1)
“I believe AI can serve as a quality control for the dentist’s diagnosis.”	4.1 (0.8)
“I would feel safer with a dentist if AI were used.”	3.1 (1.0)
“I fear that AI can make mistakes and lead to wrong decisions by the dentist.”	2.7 (0.8)
“I fear that a dentist would rely solely on AI to diagnose.”	3.0 (1.0)
“I think that some problems with my teeth could have been prevented if the dentist had worked with AI to assist.”	2.7 (1.2)
“I find it important to have a good understanding of the results of a radiograph.”	4.6 (0.7)
“I would pay something for an AI-assisted radiograph analysis.”	3.0 (1.2)
“I think it’s important to know how AI works when it is used on my radiograph.”	4.2 (0.9)
“I think it’s important that I, as a patient, am shown what is conspicuous in a radiograph.”	4.8 (0.6)
“Humans and AI can complement each other.”	4.5 (0.8)
“AI can help dentists evaluate radiographs.”	4.4 (0.7)
“In my opinion, people make more mistakes than computers.”	3.2 (0.9)

**Table 3:** Results of the logistic ordinal regression. We assessed how different covariates (age, sex, education, occupation) and psychological parameters (PHQ-4: Anxiety and Depression; BFI-10: extraversion, neuroticism, openness, conscientiousness, and tolerance as BFI-Score = BFI-S) were associated with the difference between AI-based and conventional communication on patients seeing, believing, understanding, trusting, and feeling towards the radiographic diagnosis of a caries lesion (see Figure 2). The regression analysis indicates how the covariates and the parameters modified the possible increases or decreases in these outcomes when using AI-based instead of conventional communication; the measure of this modification is the expressed as scale difference (LD) and 95% confidence intervals (CI). Bold values indicate statistical significance (*p* < 0.05). (ref. = reference).

		See		Believe		Understand		Trust		Feel Informed		Feel Unable	
Covariates and Psychological Parameters		LD	95% CI	LD	95% CI	LD	95% CI	LD	95% CI	LD	95% CI	LD	95% CI
Age group	<52 years (ref.: ≥52 years)	**1.12**	**0.38, 1.86**	0.67	−0.02, 1.37	0.61	−0.33, 1.55	−0.32	−1.93, 0.66	0.79	0.13, 1.71	−0.20	−1.05, 0.64
Sex	Female (ref.: male)	0.36	−0.34, 1.05	−0.18	−0.84, 0.48	−0.46	−1.36, 0.44	0.89	−0.09, 1.86	0.37	−0.53, 1.27	−0.26	−1.07, 0.56
Education	university degree (ref.: no university degree)	**0.90**	**0.21, 1.59**	0.55	−0.09, 1.19	0.23	−0.64, 1.10	0.15	−0.76, 1.06	0.90	−0.01, 1.80	0.50	−0.30, 1.29
Occupation	with	−0.39	−1.59, 0.81	0.16	−0.96, 1.28	−0.85	−2.40, 0.70	−0.23	−1.87, 1.40	0.61	−1.28, 2.50	0.06	−1.33, 1.44
	without	−0.56	−2.24, 1.13	0.22	−1.36, 1.82	0.14	−1.96, 0.70	−0.66	−2.94, 1.62	0.17	−2.28, 2.61	−0.00	−1.96, 1.96
	Student (ref.: retired)	1.19	−0.47, 2.85	**1.98**	**0.34, 3.62**	−1.48	−3.70, 0.72	0.18	−2.18, 2.53	1.60	−0.73, 3.94	1.68	−0.32, 3.67
Anxiety		**0.99**	**0.13, 1.86**	0.70	−0.13, 1.54	−0.91	−2.08, 0.25	−0.21	−1.43, 1.01	0.63	−0.50, 1.76	0.97	−0.08, 2.02
Depression		−0.68	−1.72, 0.37	0.01	−0.97, 0.98	−0.72	−2.10, 0.66	0.61	−2.08, 0.86	−1.19	−2.69, 0.34	0.23	−0.97, 1.42
Extraversion		0.083	−0.23, 0.45	**−0.38**	**−0.69, −0.08**	0.07	−0.34, 0.48	0.02	−0.40, 0.45	0.36	−0.07, 0.79	−0.26	−0.63, 0.11
Neuroticism		0.102	−0.24, 0.45	0.18	−0.15, 0.50	0.21	−0.24, 0.66	**0.48**	**0.01, 0.96**	0.25	−0.20, 0.69	−0.8	−0.48, 0.32
Openness		0.04	−0.36, 0.44	−0.28	−0.65, 0.11	0.31	−0.22, 0.84	0.06	−0.48, 0.60	**0.64**	**0.05, 1.24**	0.32	−0.15, 0.78
Conscientiousness		−0.87	−0.75, 0.18	−0.05	−0.49, 0.39	−0.20	−0.78, 0.38	−0.14	−0.77, 0.49	−0.18	−0.76, 0.41	0.33	−0.22, 0.88
Tolerance		0.01	−0.42, 0.45	−0.22	−0.63, 1.36	−0.13	−0.42, 0.68	−0.54	−1.12, 0.05	−0.55	−0.13, 1.71	−0.09	−0.59, 0.41

## Data Availability

Not applicable.
